# Adjunctive Dietary Therapy Is Associated With Improved Gastrointestinal Symptoms in Steroid-Refractory Gastrointestinal Graft-Versus-Host Disease: A Case Series

**DOI:** 10.1097/PG9.0000000000000203

**Published:** 2022-05-09

**Authors:** Tanyaporn Kaenkumchorn, David L. Suskind, Dale Lee, Namita Singh, Madeline Ford, Kristina Skeen, Paula C. Macris, Cecilia Yeung, Corinne Summers, Paul A. Carpenter, Hengqi B. Zheng

**Affiliations:** From the * Division of Gastroenterology, Hepatology, and Nutrition, Department of Pediatrics, Seattle Children’s Hospital, Seattle, WA; † Clinical Nutrition, Seattle Children’s Hospital, Seattle, WA; ‡ Medical Nutrition Therapy Services, Seattle Cancer Care Alliance, Seattle, WA; § Division of Clinical Research, Fred Hutchinson Cancer Research Center, Seattle, WA.

**Keywords:** graft-versus-host disease, diet, gastrointestinal, exclusive enteral nutrition, specific carbohydrate diet, pediatrics

## Abstract

Acute gastrointestinal graft-versus-host disease (GI GVHD) is a complication after hematopoietic stem cell transplant with high morbidity and mortality. In particular, steroid-refractory GI GVHD can be difficult to treat. Recent investigations have revealed that patients after transplant can experience intestinal dysbiosis contributing to the progression of GVHD. Modulation of the gut microbiome through dietary intake could potentially improve the intestinal dysbiosis in GI GVHD. In this case series, we present 3 patients where dietary therapy was used in conjunction with immunosuppression to achieve clinical remission of GI GVHD.

## INTRODUCTION

Acute graft-versus-host disease (aGVHD) after hematopoietic cell transplant (HCT) is an inflammatory condition with significant morbidity and mortality, particularly when steroid-refractory (SR-aGVHD) and involving viscera (1). Efforts to understand microbial dysbiosis in intestinal GVHD aim to develop novel treatments (2). Pediatric Crohn’s disease (pediCD) is another inflammatory gastrointestinal (GI) disease where dietary therapies like exclusive enteral nutrition (EEN) or the specific carbohydrate diet (SCD) have been successful (3,4). In patients with pediCD, EEN monotherapy was equivalent to glucocorticoids in inducing clinical remission but with superior mucosal healing (3). EEN is hypothesized to modulate intestinal dysbiosis, nourish intestinal epithelium, deliver antigenic monotony, and minimize potential deleterious dietary exposures. By eliminating grains, most dairy products and sugars, and processed foods, SCD has also been described to induce remission in pediCD (4).

We report a retrospective chart review of 3 pediatric patients who developed SR-aGVHD after total body irradiation-based myeloablative conditioning (MAC) and cord blood transplantation (CBT) between 2015 and 2020. aGVHD prophylaxis was cyclosporine and mycophenolate mofetil (MMF). They were treated with immunosuppression plus diet to induce clinical remission. The study was approved by Seattle Children’s Hospital Institutional Review Board (STUDY00000391). We expand upon a report from our institution by Zheng et al describing 2 patients with steroid-resistant GVHD who achieved clinical remission with dietary therapy in conjunction with immunosuppression (Table 1) (5).

**TABLE 1.** Patient demographics, immunosuppression, and dietary therapy

Patient	Age (y)	Neoplasm	Type of transplant	Conditioning regimen	Immunosuppressive medications	GVHD grade/GI symptoms	Dietary therapy	Concomitant use of PN
1	19	Large B-cell lymphoma	Allogenic unrelated, mismatched double CBT	MAC: Fludarabine, Cyclophosphamide, TBI	*Treatment:*Beclomethasone, Budesonide, Methylprednisolone, Prednisone, ATG (3 doses), ECP (6 months), MSC 12 doses). *Prophylaxis:*MMF, Cyclosporine, Sirolimus	**IV:** skin 2, liver 3, gut 4. *Peak Stool Output*: >1 L/day, frankly bloody *Post induction with EEN*: 1-2 formed, nonbloody bowel movements/day by patient report	EEN, mSCD, Whole foods diet (all PO)	No
2	3	Infantile ALL	Single mismatched unrelated donor CBT	MAC: Treosulfan, Fludarabine, TBI	*Treatment:*Methylprednisolone, Budesonide, Prednisolone, Ruxolitinib. *Prophylaxis:*MMF, Cyclosporine, Sirolimus.	**IV:** skin 3, liver 0, gut 4. *Peak Stool Output*: 1.4L/day (110 mL/kg/day) with mucous and blood. *Post induction with EEN:* 5 to 8 loose stools with scant blood per day by parent report	EEN via NG, SCD PO	No
3	19	AML	Mismatched CBT	MAC: Fludarabine, Cyclophosphamide, TBI	*Treatment:*Beclomethasone, Budesonide, Methylprednisolone, Prednisone, ATG (3 doses), ECP (3 months). *Prophylaxis:*MMF, Cyclosporine, Tacrolimus	**IV:** skin 2, liver 3, gut 4. *Peak Stool Output:*5 L/day (80 mL/kg/day) *Post induction with EEN:* 1–2 nonbloody bowel movements/day	EEN, SCD, Whole foods diet (all PO)	Yes
4*	9	AML	Allogenic mismatched unrelated donor CBT	MAC: Fludarabine, Cyclophosphamide, TBI	*Treatment:*Beclomethasone, Budesonide, Methylprednisolone, Prednisone, Infliximab (4 doses), MSC (8 doses). *Prophylaxis:*MMF, Cyclosporine	**IV:** skin 3, liver 2, gut 4. *Peak Stool Output:* >2L/day (53 mL/kg/day) *Post induction with EEN:* 300 mL/day (8 mL/kg/day) stool	EEN NG, SCD PO	Yes
5*	15	T-cell ALL	Allogenic unrelated, mismatched CBT	MAC: Fludarabine, Cyclophosphamide, TBI	*Treatment:*Beclomethasone, Budesonide, Methylprednisolone, Prednisone, MSC (4 doses), Ruxolitinib, ECP (9 months). *Prophylaxis:*MMF, Cyclosporine, Tacrolimus, Sirolimus	**IV:** skin 3, liver 2 gut 4. *Peak Stool Output*: 1.5 L/day (34 mL/kg/day) with blood *Post induction with EEN:* 400 mL/day (9 mL/kg/day) stool	PEN, mSCD (all PO)	Yes

ALL = acute lymphocytic leukemia; AML = acute myelogenous leukemia; ATG = antithymocyte globulin; ECP = extracorporeal photopheresis; EEN = exclusive enteral nutrition; MAC = myeloablative conditioning; MMF = mycophenolate mofetil; mSCD = modified specific carbohydrate diet; NG = nasogastric; PEN = partial enteral nutrition; PO = by mouth; SCD = specific carbohydrate diet; TBI = total body irradiation.

*These patients are included from the report from Zheng et al (^5^).

## CASE REPORT

### Patient 1

A 19-year-old female with relapsed diffuse large B-cell lymphoma developed histologically confirmed SR-aGVHD 1 month after double CBT. Peak grade IV SR-aGVHD was characterized by hematochezia (>1 L/day) treated with methylprednisolone 2 mg/kg/day, beclomethasone, and budesonide. She also received 3 doses of antithymocyte globulin (ATG), extracorporeal photopheresis (ECP), and mesenchymal stomal cells. Her course was complicated by thrombotic microangiopathy; eculizumab was given, and cyclosporine and MMF were transitioned to sirolimus.

Seven months posttransplant, due to hematochezia, she received 2 weeks of EEN (polymeric formula 1.0 kcal/mL) by mouth, then a modified SCD which allowed oats and rice for 8 weeks. Total parenteral nutrition (TPN) was not used. No changes were made to the pharmacologic regimen during the time of dietary therapy initiation. She transitioned to a whole foods diet with minimal sugars, lactose, and processed foods for 8 additional weeks. After maintaining this diet for 18 months posttransplant, she reports resolution of GI symptoms.

### Patient 2

A 2-year-old female with infant ALL developed SR-aGVHD within 1 month of unrelated donor CBT, then 7 months later peaked at grade IV with hematochezia. Treatment was with methylprednisolone 2 mg/kg/day, budesonide, prednisolone, and ruxolitinib; cyclosporine and MMF were switched to sirolimus.

Seventeen months posttransplant, she began EEN (elemental formula 1.0 kcal/mL) via nasogastric tube (NG) for a flare-up of GVHD. TPN was not used. Medical therapy was sustained during initiation of dietary therapy. After 3 weeks of EEN, symptomatic improvement prompted transition to a whole foods-based EEN formula with intact protein via NG (plant-based polymeric formula 1.2 kcal/mL) for 10 months. As stool frequency improved, EEN was transitioned to SCD that continues 2 years later. The patient experiences nausea and emesis with introduction of non-SCD foods.

### Patient 3

**FIGURE 1.**
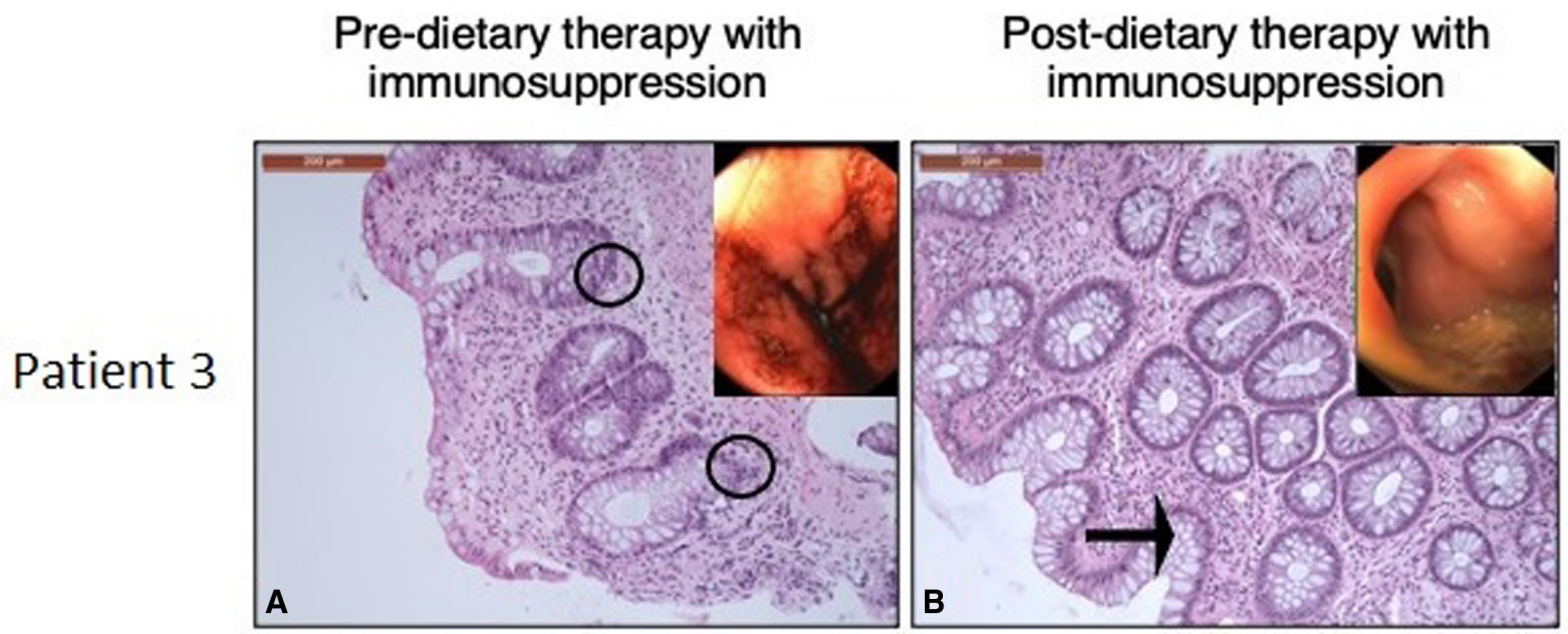
Histologic and endoscopic evaluation of patient 3, predietary and postdietary therapy. A) H&E at 10×, respectively, histologic GVHD with increased apoptosis (circles). Endoscopic colonic erythema and edema. B) H&E at 10×, respectively, no GVHD, normal appearing glands, focal colitis (arrow). Endoscopically normal colon. GVHD = graft-versus-host disease.

A 19-year-old female with therapy associated acute myelogenous leukemia (AML) developed histologically confirmed, peak grade IV SR-aGVHD (Fig. 1A) after unrelated donor CBT. SR-aGVHD was characterized by hematochezia (5 L/day) with abdominal pain. She was treated with beclomethasone, budesonide, methylprednisolone 2 mg/kg/day, prednisone, 3 doses of ATG, and ECP; cyclosporine was switched to tacrolimus.

Seven months posttransplant, she received 5 weeks of EEN (polymeric formula 1.5 kcal/mL) by mouth due to hematochezia, diarrhea, and poor intake. The patient received TPN. Immunosuppression regimen for GVHD was sustained during the start of dietary therapy. Intestinal symptoms resolved, and EEN was transitioned to SCD for 4 weeks before liberalizing to a whole foods diet with minimal sugars and processed foods. She had continued nausea and underwent endoscopy with biopsies that did not reveal GVHD but showed mild focal active/chronic inflammation of unclear significance (Fig. 1B). She eliminated dairy postprocedure with resolution of GI symptoms thereafter. Two years posttransplant, she is off immunosuppression and on a whole foods diet.

## DISCUSSION

SR-GVHD is difficult to treat despite multiple immunosuppressive therapies. Here, we describe 3 patients who benefitted from dietary therapy in conjunction with immunosuppressive therapy for treatment of GI GVHD. Zheng et al. previously described 2 patients who benefitted from dietary therapy in addition to immunosuppression in the treatment of steroid-resistant GVHD (5). This report included a 9-year-old male with AML who developed steroid-resistant grade IV GVHD who was started on EEN before transitioning to SCD. The second patient was a 15-year-old with ALL who developed grade IV GVHD, presenting with increased volume of bloody stools. He subsequently received partial enteral nutrition, full enteral nutrition, and SCD before resuming a regular diet. Both patients were able to discontinue immunosuppression.

Increasingly, studies implicate intestinal dysbiosis in the development and progression of GVHD (2). The intestinal microbiota represents a potential treatment target, as diet is a well-known modulator of the intestinal microbiome. The improvement in our patients suggests dietary therapies that facilitate mucosal regulation in pediCD such as EEN and SCD may provide clinical benefit for intestinal GVHD (3,4). These patients all had a similar trajectory of clinical improvement in GVHD once dietary therapy was added to immunosuppression.

In terms of formula type and route of administration in this patient population, there was no difference in compliance between oral and enteral intake. We found no difference in feeding tolerance otherwise. Based on this small case series, we cannot make a recommendation regarding elemental, semielemental, or nonelemental formula. Studies on different types of formula in pediCD found no difference in the type of formula (ie, elemental, semi elemental) and outcomes (6). This suggests standard polymeric formulas may be used if tolerated. Regarding palatability and tolerance to EEN by mouth, the oncologic population is highly motivated. The decision to proceed with oral feeds versus NG placement should be made through discussion with family, bone marrow transplant, and gastroenterology teams. Patient age is a key consideration in terms of formula route. Furthermore, in the oncologic population, neutropenia, thrombocytopenia, and mucositis are factors to consider before NG placement if oral feeds are not an option due to formula fatigue or poor palatability. A limitation of this series is potential confounding by concurrent immunosuppressive therapies at the time of dietary therapy. However, all patients began dietary therapy in the context of poor response to immunosuppressive therapies. Additionally, while all patients had significant symptomatic improvement, repeat endoscopic evaluation to document histological mucosal healing was not performed in all patients. Finally, it is noted that no microbiome specimens were obtained that could help corroborate the hypothesized change in microbiome due to diet.

Given the mortality and morbidity associated with SR-GVHD, future studies are needed to elucidate safe treatment options. Dietary therapy could be considered as adjunctive therapy in patients with intestinal GVHD.

## ACKNOWLEDGMENTS

We would like to thank Dr. Jeffrey B. Walker at the Boise Pathology Group at St. Luke’s in Boise, ID for histologic images.T.K. and B.Z. conceptualized and drafted the manuscript. All authors edited and approved the final manuscript. Guarantor of the article: H.B.Z.
